# Development and evaluation of a rapid RPA/CRISPR-based detection of *Francisella tularensis*

**DOI:** 10.3389/fmicb.2022.901520

**Published:** 2022-08-10

**Authors:** Jian-Hao Xu, Lin Kang, Bing Yuan, Zi-Han Feng, Shi-Qing Li, Jing Wang, Ya-Ru Wang, Wen-Wen Xin, Shan Gao, Jia-Xin Li, Yan-Song Sun, Jing-Lin Wang, Yuan Yuan

**Affiliations:** ^1^State Key Laboratory of Pathogen and Biosecurity, Beijing Institute of Microbiology and Epidemiology, Academy of Military Medical Sciences (AMMS), Beijing, China; ^2^School of Life Sciences, Fujian Agriculture and Forestry University, Fuzhou, China; ^3^Department of Disease Control and Prevention, The No. 96609 Hospital of Chinese People's Liberation Army, Yinchuan, China; ^4^Faculty of Life Science and Technology, Kunming University of Science and Technology, Kunming, China

**Keywords:** *Francisella tularensis*, recombinase polymerase amplification, CRISPR/Cas12a, rapid detection, high-sensitive

## Abstract

*Francisella tularensis* is a dangerous pathogen that causes an extremely contagious zoonosis in humans named tularemia. Given its low-dose morbidity, the potential to be fatal, and aerosol spread, it is regarded as a severe threat to public health. The US Centers for Disease Control and Prevention (CDC) has classified it as a category A potential agent for bioterrorism and a Tier 1 Select Agent. Herein, we combined recombinase polymerase amplification (RPA) with CRISPR/Cas12a system to select the *F. tularensis* target gene (TUL4), creating a two-pronged rapid and ultrasensitive diagnostic method for detecting *F. tularensis*. The real-time RPA (RT-RPA) assay detected *F. tularensis* within 10 min at a sensitivity of 5 copies/reaction, *F. tularensis* genomic DNA of 5 fg, and *F. tularensis* of 2 × 10^2^ CFU/ml; the RPA-CRISPR/Cas12a assay detects *F. tularensis* within 40 min at a sensitivity of 0.5 copies/reaction, *F. tularensis* genomic DNA of 1 fg, and *F. tularensis* of 2 CFU/ml. Furthermore, the evaluation of specificity showed that both assays were highly specific to *F. tularensis*. More importantly, in a test of prepared simulated blood and sewage samples, the RT-RPA assay results were consistent with RT-PCR assay results, and the RPA-CRISPR/Cas12a assay could detect a minute amount of *F. tularensis* genomic DNA (2.5 fg). There was no nonspecific detection with blood samples and sewage samples, giving the tests a high practical application value. For example, in on-site and epidemic areas, the RT-RPA was used for rapid screening and the RPA-CRISPR/Cas12a assay was used for more accurate diagnosis.

## Introduction

*Francisella tularensis* is a Gram-negative, facultative intracellular bacterium that can remain in water and soil environments for prolonged periods and causes tularemia in humans and many animals (Maurin and Gyuranecz, 2016; Pilo, 2018). In practice, studies have shown that only *F. tularensis* subsp. *tularensis* (Type A, in North America) and *F. tularensis* subsp. *holarctica* (Type B, found throughout the northern hemisphere) can cause tularemia (McLendon et al., 2006). Human infection is usually the result of exposure to the pathogen through contact with sick animals, Ixodidae ticks, mosquitoes, or contaminated hydro-telluric environments (Sjöstedt, 2007; Appelt et al., 2020). The ability of *F. tularensis* to spread through aerosol transmission enables it to spread rapidly in the population (Barnes et al., 2020; Maurin, 2020). Owing to its extreme infectivity and low-dose morbidity, it is regarded as a potential biological weapon, and the US Centers for Disease Control and Prevention (CDC) has classified it as a category A potential agent for bioterrorism and a Tier 1 Select Agent (Dennis et al., 2001; Euler et al., 2012). To promptly obtain appropriate treatment for patients and to avoid the rapid spread of tularemia in the population, accurate, rapid, sensitive, and easy-to-use diagnostic tools that can detect *F. tularensis* are essential.

The traditional isolation and culture of microorganisms for morphological identification are time-consuming. Moreover, it is laborious to isolate *F. tularensis* from clinical samples and the bacterium is typically obtained in <10% of patients (Afset et al., 2015; Maurin, 2020). Currently, the detection of *F. tularensis* is mainly based on serological methods (Yanes et al., 2018; Esmaeili et al., 2019). However, this diagnosis method has limitations in terms of sensitivity and specificity, often giving false positives, and thus cannot be used as a standard diagnostic method (Maurin, 2020). While PCR-based detection methods satisfy the requirements of sensitivity and specificity (Sabour et al., 2020), their use is restricted by the requirement of expensive instruments and well-trained laboratory personnel. The two requirements make applying the method in resource-poor areas and for on-site testing challenging (James and Macdonald, 2015).

Recombinase polymerase amplification (RPA) is a recently developed thermostatic amplification technology (Lobato and O'Sullivan, 2018). In an isothermal reaction condition (39–42°C), trace target DNA can be expanded to a detectable level within 10 min. Therefore, RPA is an ideal nucleic acid amplification technology that has an uncomplicated operation and inexpensive instruments (James and Macdonald, 2015; Li et al., 2020). Clustered regularly interspaced short palindromic repeats–CRISPR-associated proteins (CRISPR–Cas) is an immune defense system used by bacteria to resist exogenous DNA infection (Makarova and Koonin, 2015). Immunity is obtained by combining short fragments of exogenous DNA into CRISPR loci, followed by transcription and processing, to produce CRISPR RNAs (crRNAs) that guide Cas endonuclease proteins to complementary invading nucleic acids, resulting in target interference (van der Oost et al., 2014). The Class II Cas protein Cas9 has been widely used in gene editing and transcription regulation research (Boutin et al., 2021; Moreb and Lynch, 2021). Recently, Cas13a and Cas12a effectors have also shown promise for new research directions in nucleic acids detection as they have the unique property of trans-cleavage of single-stranded non-target RNA (Cas13a) or DNA (Cas12a) (Strohkendl et al., 2018; Barnes et al., 2020). Typically, a CRISPR-based sensing platform is combined with pre-amplification processes such as RPA and loop-mediated isothermal amplification (LAMP) to efficiently improve analytical performance. For example, the Doudna lab combined RPA with the CRISPR/Cas12a platform to develop a technique for rapid and specific detection of human papillomavirus (Chen et al., 2018), and Broughton et al. combined RT-LAMP with the CRISPR/Cas12a platform to effectively detect SARS-CoV-2 (Broughton et al., 2020).

**Figure 1:**
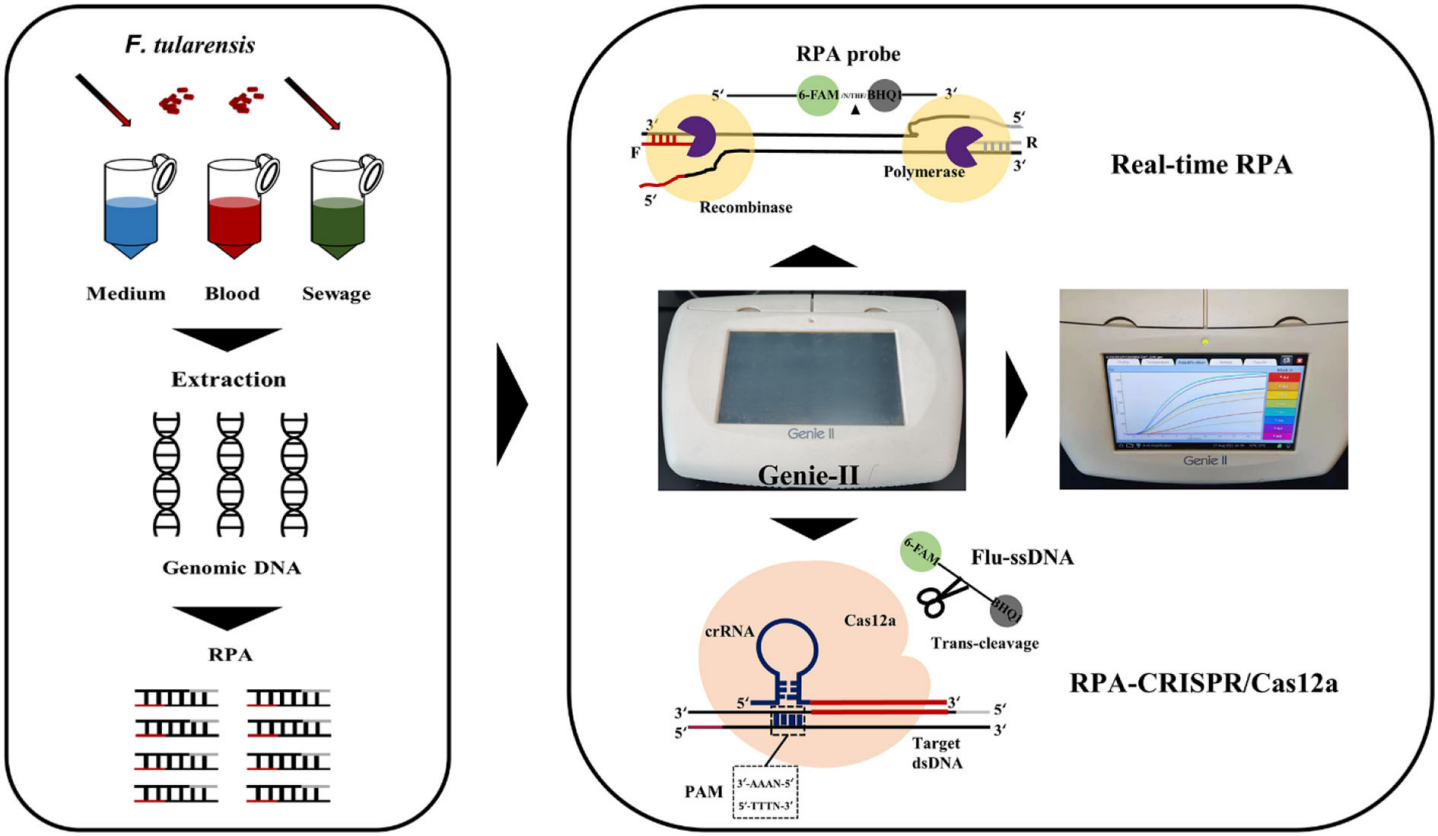
Schema of assay process for the detection of *F. tularensis* with RT-RPA and RPA-CRISPR/Cas12a assays.

In this study, we developed a real-time RPA (RT-RPA) and RPA-CRISPR/Cas12 assays of *F. tularensis* with the target gene TUL4, respectively (Figure 1). The RT-RPA assay takes 10 min, and the RPA-CRISPR/Cas12 assay takes 40 min. The limits of detection are 5 copies/reaction and 0.5 copies/reaction for RT-RPA and RPA-CRISPR/Cas12, respectively. We used the compact portable Genie-II instrument (OptiGene, UK) in this study. The Genie-II is equipped with a lithium battery and can keep working for 12 h in the field. The RPA/CRISPR-based detection of *F. tularensis* established in this study is rapid, ultrasensitive, and specific, which might promise a broad prospect in early clinical diagnosis, biosafety prevention, and other studies involving *F. tularensis*.

## Materials and methods

### Materials

The following bacterial strains were available in our lab: *F. tularensis, Brucella melitensis, Brucella abortus, Burkholderia pseudomallei, Burkholderia mallei, Bacillus anthracis, Staphylococcus aureus, Bacillus thuringiensis, Yersinia pestis, Salmonella typhi, Bacillus subtilis, Escherichia coli, Vibrio vulnificus, Staphylococcus epidermidis, Vibrio parahaemolyticus, Bacillus cereus*, and *Vibrio cholerae*. RPA primers, RPA probes, crRNA, and fluorescent single-stranded DNA reporter (Flu-ssDNA) were synthesized by Shanghai Sangon Biotech Co., Ltd. (China). LbCas12a protein, NEBuffer 3.1, and RNA inhibitor were provided by New England BioLabs, Inc. (USA). TwistAmp™ Exo Kit and TwistAmp™ basic Kit were provided by TwistDx (Cambridge, UK). A positive reference plasmid for *F. tularensis* detection (pEASY-T1-TUL4) was constructed by our lab. DNase/RNase-free distilled, deionized water (DDH_2_O) was provided by Tiangen Biochemical Co., Ltd. A QIAamp™ DNA Mini Kit (Qiagen, Germany) was used to extract bacterial strain genomes, followed by the user manual's protocol.

### The heat-inactivated bacteria and genomic DNA preparation

The heat-inactivated *B. melitensis, B. abortus, B. pseudomallei, B. mallei, B. anthracis, Y. pestis*, and *F. tularensis* were prepared in the BSL-3 lab. Specifically, these bacteria were cultured in BSL-3, which subsequently were serially diluted and plated on Francis agar or Columbia blood agar. After incubation, the number of CFU was determined as CFU/ml. The serially diluted bacteria are inactivated by heat. Subsequently, these heat-inactivated bacteria were taken out of BSL-3 and used for genomic DNA extraction. *S. aureus, B. thuringiensis, S. typhi, B. subtilis, E. coli, V. vulnificus, S. epidermidis, V. parahaemolyticus, B. cereus*, and *V. cholerae* were cultured in BSL-2 and also inactivated by heat. The QIAamp™ DNA Mini Kit (Qiagen, Germany) was used to extract genomic DNA from all these bacteria. For *F. tularensis* experiments, *F. tularensis* was cultured on a Francis agar plate at 37°C. Then, phosphate-buffered saline (PBS) was used to wash and collect these colonies. After 10-fold serial dilution, 100 μl of each gradient diluent was coated on three parallel Francis agar. The concentration of the original bacterial solution (CFU/ml) = N × M × 10 (N = mean of colonies counts on three plates; M = dilution multiple). During the RT-RPA assay and RPA-CRISPR/Cas12a assay, the inactivated bacterial solution was diluted to 2 × 10^3^, 2 × 10^2^, 2 × 10^1^, and 2 × 10^0^ CFU/ml, and then QIAamp™ DNA Mini Kit (Qiagen, Germany) were used to extract nucleic acid and 2 μl of nucleic acid as template DNA for detection.

### Design of RPA primers, RPA probes, and CrRNA

The 17 kDa major membrane protein-encoding gene TUL4 of *F. tularensis* was used as the primary target gene (GenBank: M32059.1). The complete sequence of the gene TUL4 was constructed into a positive reference recombinant plasmid (pEASY-T1-TUL4), whose total sequence length was 4,865 bp.

According to the assay design manual of the TwistAmp™ DNA amplification kits, RPA primers tul-F1/R1 and tul-F2/R2 were designed by Primer Premier 6. RPA probes tul-P1 and tul-P2 were designed by the amplified sequence of tul-F1/R1 and tul-F2/R2, respectively. Details of oligonucleotides are listed in Table 1.

**Table 1:** The involved oligonucleotides sequence.

**Name**	**Sequence (5^′^-3^′^)**
tul-F1	GTCATCTTGATCTTATCTTAGCGACTAATCCT
tul-R1	TATATGTCTTACAAGCAGTATCACTCGCCATA
tul-P1	GAAAAACAACTTTTGCCTCCACTTGAGATAAT(FAM-dT)A(THF)(BHQ1dT)CAAATCGCAAAAGCTG[C3 Spacer]
tul-F2	CAGCTACTACTGAGCAAGCTGCTGCTGTATCT
tul-R2	CACTTAGAACCTTCTGGAGCCTGCCATTGTAATC
tul-P2	AATAAAAGCAACTGTATATACAGCATACAA(FAM-dT)A(THF)(BHQ1dT)AACCCACAAGGAAGT[C3-Spacer]
crRNA-tul-1	UAAUUUCUACUAAGUGUAGAU CCUCCACUUGAGAUAAUUAAUCAA
crRNA-tul-2	UAAUUUCUACUAAGUGUAGAU AAUAAACUUGGUCAGGAUAAAAUA
Flu-ssDNA	FAM-CCCCCCCCCCCC-BHQ1

The crRNA spacer sequences were designed downstream of the protospacer adjacent motif (PAM) sequence containing 5′-TTTN-3′ on the RPA amplified sequence, and the anchor sequence was added upstream of the crRNA spacer sequences (Li et al., 2018). Two crRNAs, namely, crRNA-tul-1 and crRNA-tul-2, were designed by the amplified sequence of tul-F1/R1 and tul-F2/R2, respectively. Subsequently, the fluorescent single-stranded DNA reporter (Flu-ssDNA) modified with fluorophore 6-FAM, and quencher BHQ1 was trans-cleaved by Cas12a and indicated the presence or absence of the target gene TUL4. Details of oligonucleotides are listed in Table 1.

### Real-time RPA assay

The RT-RPA assay was conducted with a TwistAmp™ Exo Kit with minor changes to the reaction mix: 29.5 μl of primer-free rehydration buffer, 2.1 μl of forward primer (10 μM), 2.1 μl of reverse primer (10 μM), 0.6 μl of probe (10 μM), 3 μl of MgOAc, and 2 μl of template DNA and DDH_2_O to bring the total volume to 50 μl. The pre-matching reaction system was mixed in an Eppendorf tube and then incubated at 39°C for 10 min in the Genie-II.

### RPA-CRISPR/Cas12a assay

The primers used in the RPA-CRISPR/Cas12a assay were consistent with those in the RT-RPA assay. Template DNA was amplified by RPA to get RPA products, according to TwistAmp™ Basic Kit Quick Guide, using the following reaction mix: 29.5 μl of primer-free rehydration buffer, 2.4 μl of forward primer (10 μM), 2.4 μl of reverse primer (10 μM), 3 μl of MgOAc, and 2 μl of template DNA and DDH_2_O to bring the total volume to 50 μl. The pre-matching reaction system was mixed in a tube and then incubated at 39°C for 30 min in Genie-II.

Recombinase polymerase amplification products were then immediately added to the CRISPR/Cas12a system, which following previously published methods (Li et al., 2018), with the following optimizations for the reaction mix: 75 nM of LbCas12a, 500 nM of crRNA, 500 nM of Flu-ssDNA, 10 U of RNA inhibitor, 1.5 μl of NEBuffer 3.1, 5 μl of RPA products, and DDH_2_O supplemented to 20 μl. This pre-matching reaction system was mixed in a tube and incubated at 45°C for 10 min in the Genie-II.

### Sewage samples and blood samples test by RT-RPA, RPA-CRISPR/Cas12a, and RT-PCR assays

Whole *F. tularensis* bacteria are dangerous and must be handled under biosafety level 3 (BSL-3) conditions (Manual of Clinical Microbiology, 8th ed. American Society for Microbiology, Washington, D.C.). Therefore, we used only genomic DNA to prepare test samples.

A real-time PCR (RT-PCR) assay was used as the standard method to detect *F. tularensis* (Versage et al., 2003), which was also based on the TUL4 gene: Tul4F (5′-ATTACAATGGCAGGCTCCAGA-3′), Tul4R (5′-TGCCCAAGTTTT ATCGTTCTTCT-3′), and Tul4P (5′-6-FAM-TTCTAAGTGCCATGATACAAGCTTCCCAATTAC TAAG-BHQ1-3′).

Blood samples were prepared with fresh human whole blood provided by volunteers, and the sewage sample was composed of natural water to which sludge was added (Yuyuantan, Yongding River Basin, Beijing). First, researcher A added the gradient dilution of *F. tularensis* genomic DNA (1,250–1.25 fg/μl) to blood and sewage samples, using DDH_2_O as a blank control (BC) and randomly numbering all samples. Then, researchers B and C got unknown samples and used QIAamp™ DNA Mini Kit to extract nucleic acids from all samples. Finally, researchers B and C then tested genomic DNA extracts from all samples using RT-RPA, RPA-CRISPR/Cas12a, and RT-PCR assays. In addition, instead of nucleic acid extraction, 8 blood samples were centrifuged and the 2 μl of supernatants were used directly for RT-RPA, RPA-CRISPR/Cas12a, and RT-PCR detection.

## Results

### Optimal RPA primers/probe and sensitivity evaluation of RT-RPA assay

**Figure 2:**
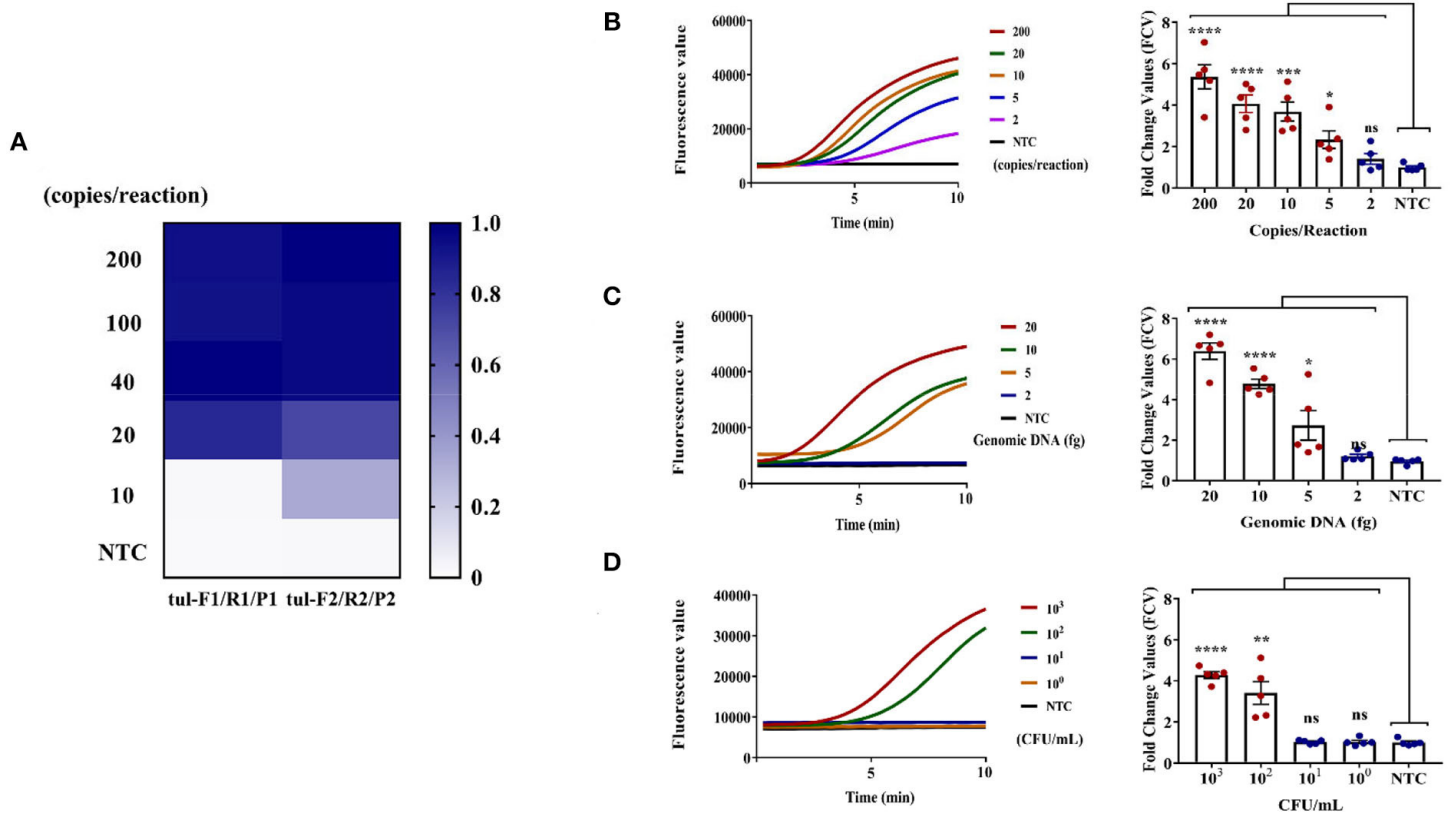
RT-RPA assay for *F. tularensis*. **(A)** Screening optima RPA primers and probe under the same DNA template gradient concentration. **(B)** Sensitivity assessment using pEASY-T1-TUL4 plasmid (2 μl of gradient concentrations of plasmid 100, 10, 5, 2.5, and 1 copies/μl), which can reach 5 copies/reaction, **** (*P* < 0.0001), *** (*P* = 0.0004), * (*P* = 0.0145). **(C)** Sensitivity assessment using genomic DNA of *F. tularensis* (2 μl of *F. tularensis* genomic DNA 10, 5, 2.5, and 1 fg/μl), which reaches 5 fg/reaction, **** (*P* < 0.0001), * (*P* = 0.0427), ns (non-significant). **(D)** Sensitivity assessment using *F. tularensis* (2 μl extracted DNA from 200 μl heat-inactivated *F. tularensis* 2 × 10^3^, 2 × 10^2^, 2 × 10^1^, 2 × 10^0^ CFU/ml), which can reach 2 × 10^2^ CFU/ml, **** (*P* < 0.0001), ** (*P* = 0.0024), ns (non-significant). The data are presented as the mean ± SEM (*n* = 5). *N* = 5 technical replicates; unpaired 2-tailed *t*-test was used to analyze the difference from the NTC. The fluorescent value–time curves of RT-RPA assay presented are from one representative experiment (the right graph of **B–D**).

Initially, we diluted the constructed pEASY-T1-TUL4 with DDH_2_O, which was stored at 4°C for a short time and at −40°C for a long time. The gradient concentration of pEASY-T1-TUL4 (ranging from 200 to 10 copies/reaction) was set as template DNA to screen tul-F1/R1/P1 and tul-F2/R2/P2 using an RT-RPA assay; at the same time, DDH_2_O was set to non-template control (NTC). The fluorescent signal of each group after 10 min of reaction time was taken and normalized to make a heat map (Figure 2A). As shown in the figure, the RT-RPA assay guided by tul-F2/R2/P2 could detect template DNA with a concentration of 10 copies/reaction, consequently selecting it as the optimal RPA primer probe.

After identifying the best RPA primer/probe, the sensitivity of the RT-RPA assay was evaluated. Notably, 2 μl of gradient concentrations of positive plasmid pEASY-T1-TUL4 (100, 10, 5, 2.5, and 1 copies/μl), 2 μl of *F. tularensis* genomic DNA (10, 5, 2.5, and 1 fg/μl), and 2 μl extracted DNA from 200 μl heat-inactivated *F. tularensis* (2 × 10^3^, 2 × 10^2^, 2 × 10^1^, and 2 × 10^0^ CFU/ml) were used to evaluate the sensitivity of the RT-RPA assay, again using DDH_2_O as the NTC in the meantime. A reaction time of 10 min was selected to record the fluorescent signal of each group of samples. The fluorescent signal of NTC was used to normalize the signal of each group of samples and generate the corresponding fold change values (FCVs). As shown in the left graph of Figures 2B–D, the fluorescence value–time curve showed that RT-RPA had strong positive signals at 5–10 min distinguishable from the NTC, indicating high efficiency. The right graph of Figures 2B–D showed that the sensitivity of the RT-RPA assay could reach pEASY-T1-TUL4 of 5 copies/reaction, *F. tularensis* genomic DNA of 5 fg, and *F. tularensis* of 2 × 10^2^ CFU/ml. The RT-RPA assay can detect target DNA accurately in 5–10 min and has good sensitivity.

### Optimal CrRNA and sensitivity evaluation of RPA-CRISPR/Cas12a assay

After the completion of the RT-RPA assay, crRNA was designed with the same RPA sequence to construct the CRISPR/Cas12a system, and RPA was used as a pre-amplification to establish the RPA-CRISPR/Cas12a assay.

**Figure 3:**
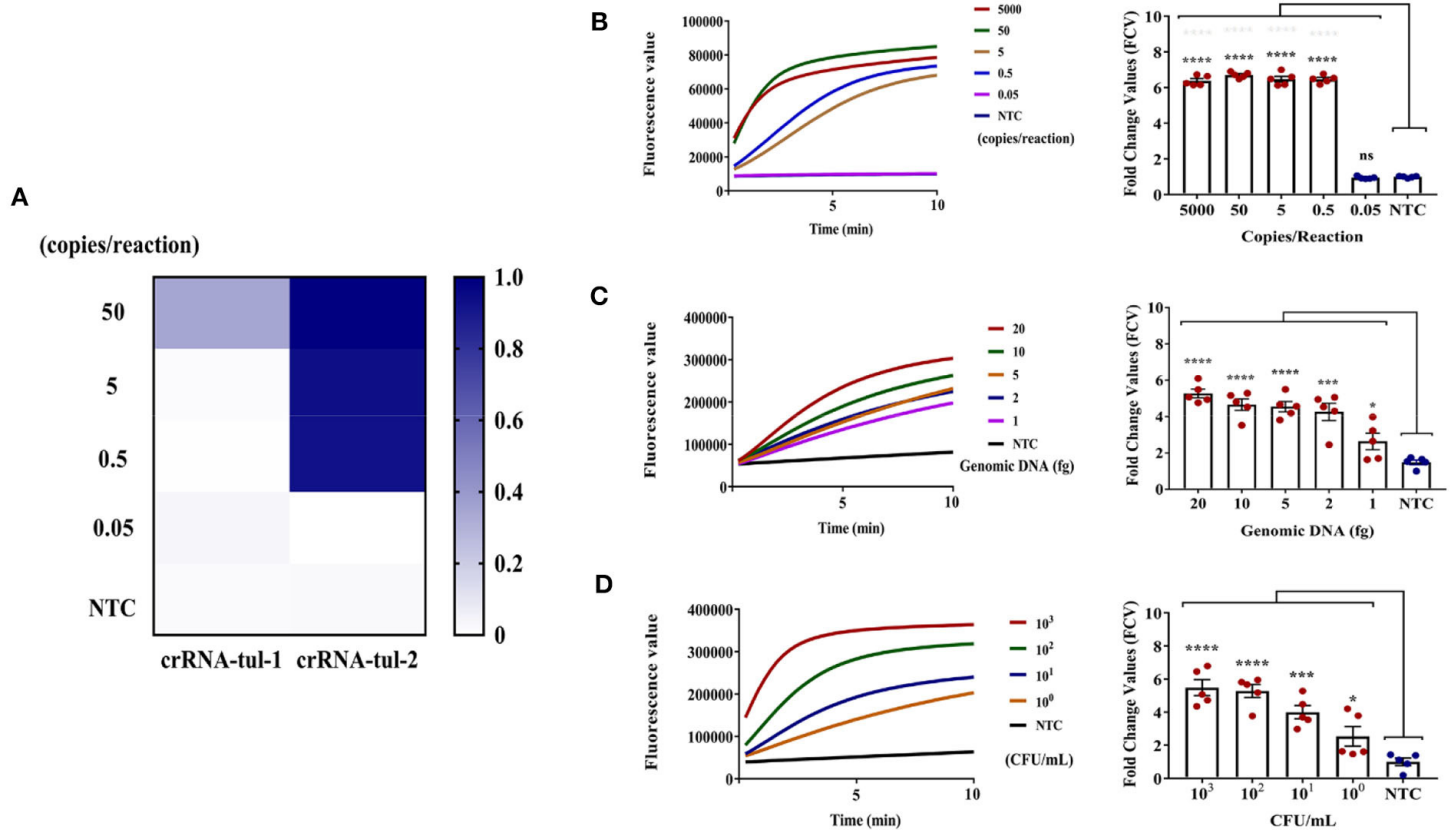
RPA-CRISPR/Cas12a assay for *F. tularensis*. **(A)** Screening optima crRNA of RPA-CRISPR/Cas12a systems under the same DNA template gradient concentration. **(B)** Sensitivity evaluation by pEASY-T1-TUL4 plasmid, which can reach 0.5 copies/reaction, **** (*P* < 0.0001), ns (non-significant). **(C)** Sensitivity evaluation using genomic DNA of *F. tularensis*, which can reach 1 fg, **** (*P* < 0.0001), *** (*P* = 0.0005), * (*P* = 0.0385), ns (non-significant). **(D)** Sensitivity evaluation using *F. tularensis*, which can reach 2 x 10^0^ CFU/ml, **** (*P* < 0.0001), *** (*P* = 0.0002), * (*P* = 0.0427). *n* = 5 technical replicates; unpaired 2-tailed *t*-test was used to analyze the difference from the NTC. The fluorescent value–time curves of RT-RPA assay presented are from one representative experiment (the right graph of **B–D**).

Initially, the pEASY-T1-TUL4 was gradiently diluted by DDH_2_O (from 5,000 to 0.05 copies/reaction) and stored at 4°C. Due to the dissimilar sequences of each crRNA, the formation efficiency of crRNA-Cas12a-target DNA ternary complexes differs, which may affect the trans-cleavage activity (Creutzburg et al., 2020). The gradient concentration of pEASY-T1-TUL4 was set as the template DNA to screen both tul-F1/R1_crRNA-tul-1 and tul-F2/R2_crRNA-tul-2 using the RPA-CRISPR/Cas12a assay, and DDH_2_O was set as the NTC. The heat map in Figure 3A showed that the sensitivity of CRISPR/Cas12a mediated by crRNA-tul-2 was significantly higher than that of crRNA-tul-1. Consequently, we selected crRNA-tul-2 as the optimal crRNA.

We next evaluated the sensitivity of the RPA-CRISPR/Cas12a assay using the gradient concentration of pEASY-T1-TUL4 (5,000, 50, 5, 0.5, and 0.05 copies/reaction), *F. tularensis* genomic DNA (20, 10, 5, 2, and 1 fg), and *F. tularensis* (2 × 10^3^, 2 × 10^2^, 2 × 10^1^, and 2 × 10^0^ CFU/ml). In the left graph of Figures 3B–D, the fluorescent value–time curve showed that the RPA-CRISPR/Cas12a assay had a clear positive signal within 40 min (RPA 30 min, CRISPR/Cas12a assay 1 0 min). The results showed that the sensitivity of the RPA-CRISPR/Cas12a assay could reach pEASY-T1-TUL4 of 0.5 copies/reaction (Figure 3B), *F. tularensis* genomic DNA of 1 fg (Figure 3C), and *F. tularensis* of 2 × 10^0^ CFU/ml (Figure 3D), indicating that this method was ultrasensitive.

### Specificity evaluation of RT-RPA and RPA-CRISPR/Cas12a assays

We verified the specificity of the RT-RPA assay and RPA-CRISPR/Cas12a assay in two ways. On the one hand, the conservative sequences of RPA primers, probes, and crRNAs in this study were evaluated by sequence alignment with *F. tularensis* subspecies and close subspecies. Furthermore, the specificity of two assays in this study was evaluated with the genomic DNA of various pathogenic bacteria in our laboratory.

**Figure 4:**
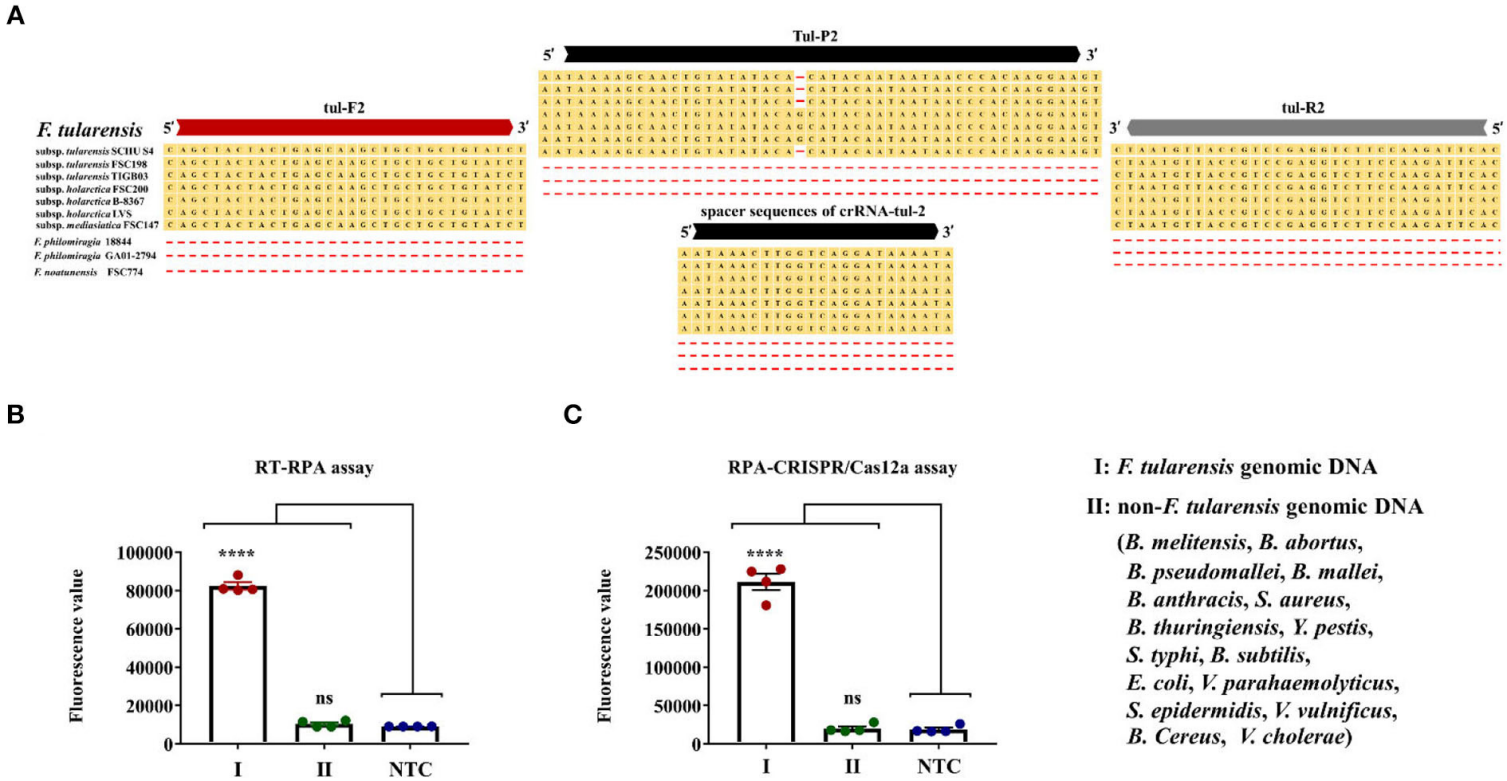
Specificity evaluation of RT-RPA and RPA-CRISPR/Cas12a assays. **(A)** Sequence alignment of tul-F2/R2, tul-P, and spacers sequence of crRNA-tul-2 with multiple *F. tularensis* strains and *F. philomiragia* and *F. noatunensis*. **(B)** Specificity evaluation of RT-RPA assay. **(C)** Specificity evaluation of the RPA-CRISPR/Cas12a assay. Error bars represent mean ± SEM, where *n* = 4 replicates, *t*-test, **** (*P* < 0.0001), ns (non-significant).

First, the complete genome sequences of several *F. tularensis* strains were downloaded from the National Center for Biotechnology Information (NCBI) and were compared with the sequences of tul-F2/R2 and crRNA-tul-2. As shown in Figure 4A, three strains of *F. tularensis* subsp. *tularensis* SCHU S4 (GCA_000008985.1), FSC 198 (GCA_000009325.1), and TIGB039 (GCA_000248415.2), three strains of *F. tularensis* subsp. *holarctica* FSC200 (GCA_000168775.2), B-8367 (GCA_010232785.1), and LVS (GCA_000833335.1), a strain of *F. tularensis* subsp. *mediasiatica* FSC147 (GCA_000018925.1), two strains of *Francisella philomiragia* 18844 (GCA_018135955.1), and GA01-2794 (GCA_000833255.1), and a strain of *Francisella noatunensis* FSC774 (GCA_014844275.1) were compared with tul-F2/R2, tul-P2, and the spacer sequences of crRNA-tul-2. Only the tul-P2 had one base mismatch when compared with *F. tularensis* subsp. *tularensis* strains, indicating that the RT-RPA and RPA-CRISPR/Cas12a assays developed in this study would detect *F. tularensis* subsp. *tularensis, F. tularensis* subsp. *holarctica*, and *F. tularensis* subsp. *mediasiatica*.

Second, we set the *F. tularensis* genomic DNA and non-*F. tularensis* genomic DNA as template DNA and DDH_2_O to NTC synchronously. The non-*F. tularensis* genomic DNA was composed of 16 bacterial genomic DNAs, including *B. melitensis, B. abortus, B. pseudomallei, B. mallei, B. anthracis, S. aureus, B. thuringiensis, Y. pestis, S. typhi, B. subtilis, E. coli, V. vulnificus, S. epidermidis, V. parahaemolyticus, B. cereus*, and *V. cholerae*. The template DNAs and NTC were tested by RT-RPA and RPA-CRISPR/Cas12a assays. Notably, the concentration of *F. tularensis* genomic DNA (25 fg/μl) was 100 times lower than that of the non-*F. tularensis* genomic DNA (2.5 pg/μl). Results of RT-RPA and RPA-CRISPR/Cas12a assays are shown in Figures 4B,C. In our independent repeated experiments, positive signals were observed only for *F. tularensis* genomic DNA, while non-*F. tularensis* genomic DNA was all consistent with the NTC results. Thus, RT-RPA and RPA-CRISPR/Cas12a assays had excellent specificity.

### Sewage samples and blood samples test by RT-RPA, RPA-CRISPR/Cas12a, and RT-PCR assays

After completing the sensitivity and specificity evaluation, we analyzed the clinical adaptation feasibility of the two methods. Since tularaemia is not common, the threat of *F. tularensis* is more likely to be a potential biological warfare agent. In this study, clinical and environmental samples were selected to be simulated samples. The clinical samples picked human blood. Considering that *F. tularensis* will spread in aerosol form and can survive in water and soil environments (Telford and Goethert, 2020; Brunet et al., 2021; Golovliov et al., 2021), we finally decided to use sludge mixed with natural water to prepare sewage samples.

**Figure 5:**
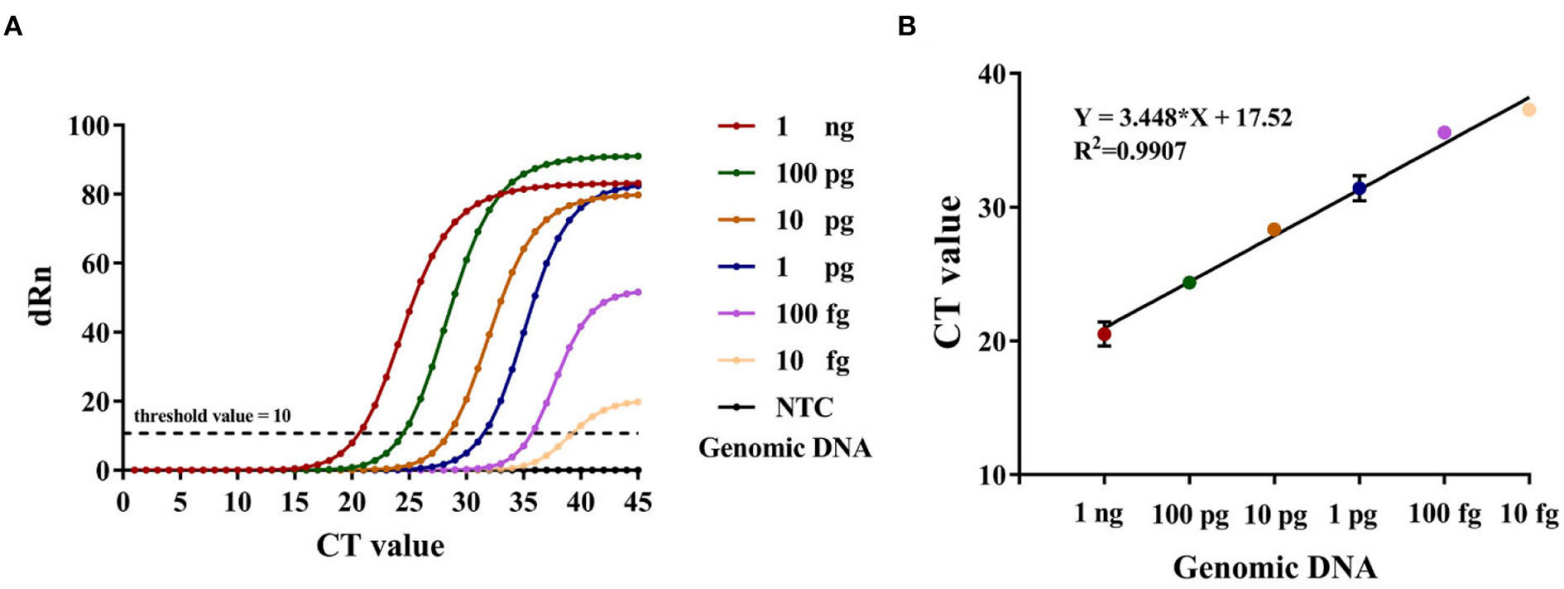
**(A)** The amplification curve of RT-PCR, Y-axis: dRn = Rn (sample) - Rn (Blank), Rn = R/RROX. **(B)** Standard curve developed using 10-fold serial dilution of DNA from *F. tularensis* ranging from 1 ng to 10 fg of genomic DNA. Error bars represent mean ± SEM, where *n* = 2 replicates.

Concomitantly, RT-PCR was used as an auxiliary reference experiment. We analyzed the feasibility of the RT-PCR assay and used it for the quantification of the genomic DNA spiked into the simulated samples. The *F. tularensis* genomic DNA was gradiently diluted by DDH_2_O (1 ng, 100, 10, 1 pg, 100, and 10 fg) and used as template DNA to make the standard curve of the RT-PCR assay, while the DDH_2_O was set to NTC synchronously (Figure 5), *R*^2^ > 0.99, *Y* = 3.448 ^*^ *X* + 17.52.

**Figure 6:**
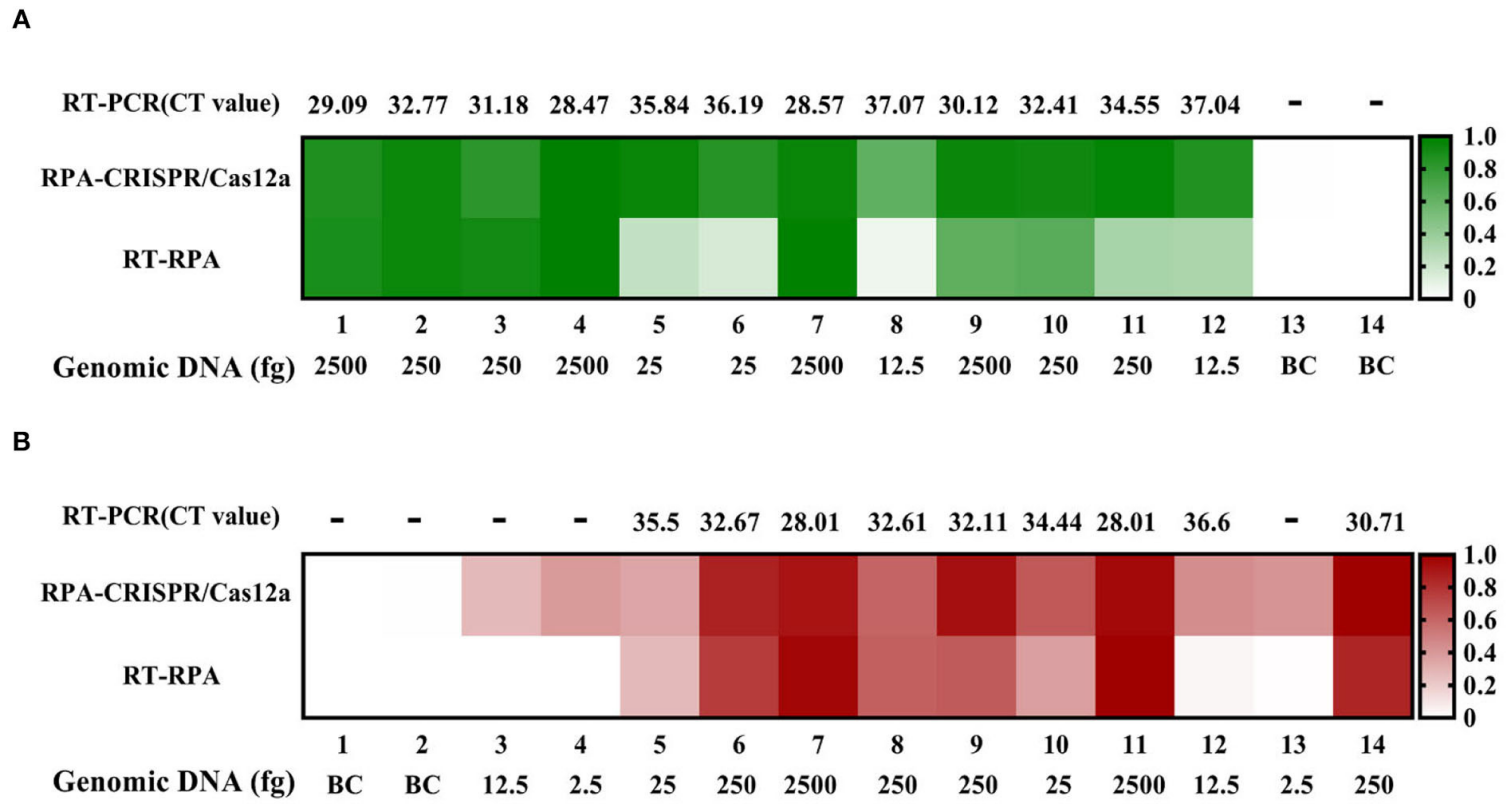
Sewage and blood sample tests by RT-RPA, RPA-CRISPR/Cas12a, and RT-PCR assays. **(A)** Sewage samples test. **(B)** Blood samples test.

Then, we prepared 14 blood samples and 14 sewage samples to evaluate the practicability of the two methods, running comparative RT-PCR assays at the same time. The RPA-CRISPR/Cas12a assay effectively detected all of the positive sewage samples. However, the RT-RPA assay failed to detect the No. 8 positive sample, and the CT values of No. 8 and 12 positive samples were both >37 in RT-PCR. Given the test results of the sewage samples, we reduced the *F. tularensis* genomic DNA spiked into blood samples. As shown in Figure 6B, there were significant differences among the assays. The RPA-CRISPR/Cas12a assay effectively detected all positive samples, while the RT-RPA assay and RT-PCR assay failed to detect No. 3, 4, and 13 positive samples, presumably due to sensitivity limitations. In addition, some samples with low nucleic acid input had discrepant RT-RPA results: No. 8 (–) and 12 (+) in sewage samples, No. 3 (–) and 12 (–) in blood samples, and this may be attributable to the low extraction efficiency of small quantities of target DNA spiked in complex samples. No false positives were detected among the 4 negative samples (BC) with these three assays (Figure 6). The results indicated that the RT-RPA assay and RT-PCR assay had a consistent test effect, while the RPA-CRISPR/Cas12a assay had a higher sensitivity than RT-RPA and RT-PCR assays.

**Figure 7:**
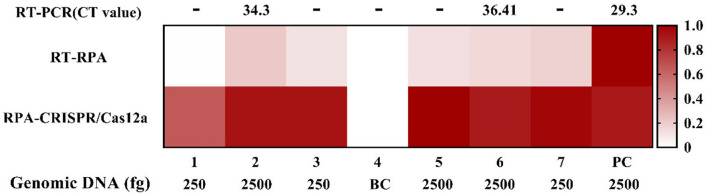
The blood sample without genomic DNA extraction tests by RT-RPA, RPA-CRISPR/Cas12a, and RT-PCR assays.

In addition, the extraction of sample nucleic acid will prolong the detection time, which is not conducive to rapid detection in the field. Therefore, we prepared the simulated blood samples without genomic DNA extraction. We used the RT-RPA assay, RPA-CRISPR/Cas12a assay, and RT-PCR to directly detect the simulated blood samples, respectively, and the results are shown in Figure 7. Without genomic DNA extraction, the sensitivity of each detection method was affected. Compared with the positive control (PC), the results of the RPA-CRISPR/Cas12a assay were not interfered with blood samples, and the detection signals of the RT-RPA assay and RT-PCR (fluorescence value and CT value) were significantly reduced. However, samples No. 3, 5, and 7 showed that the RT-RPA assay could still detect the low concentration target in blood samples.

## Discussion

*F. tularensis* is a dangerous pathogen that can infect humans, more than 190 mammalian species, and arthropods, especially ticks and mosquitoes. In humans, it causes contagious zoonosis tularemia (Hennebique et al., 2019; Zellner and Huntley, 2019). Tularemia has complex epidemiology and ecology that is regionally specific, making it difficult to be diagnosed based on clinical symptoms and delaying patient treatment (Frischknecht et al., 2019; Maurin, 2020). In addition, its high infectivity and morbidity, easy cultivation, absence of a human vaccine, and aerosol propagation make *F. tularensis* a potential biological weapon (Prokšová et al., 2019).

Traditional microbial culture and identification are time-consuming and are thus not conducive to rapid screening and early clinical diagnosis of *F. tularensis* (Afset et al., 2015). Serological testing has limited sensitivity and specificity and cannot be standardized (Maurin, 2020). RT-PCR can meet general laboratory testing needs; however, due to the need for a temperature change module, the instrument is expensive and large (James and Macdonald, 2015), which is not suitable for on-site and epidemic area detection. To counter these challenges, we developed RT-RPA and RPA-CRISPR/Cas12a assays for rapid, sensitive, and specific detection of *F. tularensis* (Figure 1). The RT-RPA assay has high detection efficiency, could rapidly detect *F. tularensis* within 10 min, and has a detection sensitivity of 5 copies/reaction, genomic DNA of 5 fg, and *F. tularensis* of 2 × 10^2^ CFU/ml (Figures 2B–D). The ultrasensitive RPA-CRISPR/Cas12a assay could rapidly detect *F. tularensis* within 40 min, and the sensitivity was pEASY-T1-TUL4 of 0.5 copies/reaction, *F. tularensis* genomic DNA of 1 fg, and *F. tularensis* of 2 × 10^0^ CFU/ml (Figures 3B–D).

TUL4, a 17-kDa lipoprotein, is one of several membrane proteins that induce an *in vitro* response in T cells from *F. tularensis*-primed humans (Sjöstedt et al., 1991). We selected the encoding gene of TUL4 as a target sequence for the RPA primers, probes, and crRNA of RT-RPA and RPA-CRISPR/Cas12a assays and then, through sequence alignment, confirmed that they are highly conservative to *F. tularensis* subsp. *tularensis* and *F. tularensis* subsp. *holarctica* (Figure 4A). The evaluation of specificity showed positive signals only for *F. tularensis* and not for any other bacteria tested, indicating that the RT-RPA and RPA-CRISPR/Cas12a assays are highly specific to *F. tularensis* (Figures 4B,C).

Furthermore, we evaluated the feasibility of the RT-RPA and RPA-CRISPR/Cas12a assays in clinical and environmental diagnosis. The results of a comparative test with RT-PCR in blood samples and sewage samples (Figures 6A,B) demonstrated that RT-RPA and RPA-CRISPR/Cas12a assays have great potential in applied settings. To meet the needs of on-site detection, we tried the nucleic acid-free extraction method for detection. Compared with nucleic acid extraction (PC), the sensitivity of each detection method was affected, but the results of the RPA-CRISPR/Cas12a assay did not interfere with blood samples. Some additional work will be necessary to fully translate this work into a widely available point-of-care device in the future. An extraction-free protocol for samples needs to minimize the number of steps in the assay.

In summary, we have developed rapid, ultrasensitive, and specific detection methods for *F. tularensis* using RT-RPA and RPA-CRISPR/Cas12a assays. Compared with other equally sensitive test assays already reported (Table 2) (Versage et al., 2003; Chen et al., 2021; Li et al., 2021; Lee and Oh, 2022), the RPA/CRISPR-based detection of *F. tularensis* in this study offers a number of advantages, namely, rapid, ultrasensitive, specific, portative, and mild; more importantly, the dually diagnostic methods for *F. tularensis* can avoid error and missed detection to a greater extent. If it is combined with the nucleic acid-free extraction method, this detection method has a great potential value in clinical rapid diagnosis and biosafety maintenance, especially for on-site diagnosis in the future.

**Table 2:** Comparison of the RPA/CRISPR-based detection of F. *tularensis* with other equally sensitive test assays already reported.

	**In the present study**			**RAA-Cas12a**	**LAMP-Cas12a**	**PCR-Cas12a**
**Target pathogen**	***F. tularensis***	***F. tularensis***	***F. tularensis***	***Listeria monocytogenes***	***Escherichia coli* O157:H7**	***Yersinia pestis***
Diagnostic strategy	Real-time RPA	RPA-CRISPR/Cas12a	Real-time PCR	RAA-Cas12a	LAMP-Cas12a	PCR-Cas12a
LoD	5 copies/reaction, 5fg of genomic DNA, 2 × 10^2^ CFU/ml	0.5 copies/reaction, 1fg of genomic DNA, 2 × 10^0^ CFU/ml	10^0^ CFU, 1 Genomic Equivalents	0.68 aM of genomic DNA; 26 CFU/ml of *L. Monocytogenes*	1.22 CFU/ml of *E. coli* O157:H7	10^3^ fg per μl input
Assay reaction time (approximate) and Components	10 min (RPA 39°C 10 min)	40 min (RPA 39°C 30 min, CRISPR/Cas12a 45°C 10 min)	47 min (50°C for 2 min, 95°C for 10 min, 45 cycles at 95°C for 10 s and 60°C for 30 s, 45°C for 5 min)	75 min (RAA 37°C 30 min, CRISPR/Cas12a 37°C 45 min)	45 min (LAMP 58.8°C 40 min, CRISPR/Cas12a 37°C 5 min)	115 min (PCR 55 min 94°C 5 min; 30 cycles of 94°C 30 s, 55°C 30 s, 72°C 30 s; 72°C 5 min. CRISPR/Cas12a 37°C 60 min)
Assay results	Qualitative	Qualitative	Quantitative	Quantitative	Qualitative	Qualitative
Bulky instrumentation Required	No	No	Yes	No	No	Yes

## Data availability statement

The original contributions presented in the study are included in the article/supplementary material, further inquiries can be directed to the corresponding author/s.

## Author contributions

Y-SS, J-LW, and YY: conceptualization, writing—review and editing, project administration, and funding acquisition. J-LW, J-HX, and YY: methodology. LK, Z-HF, and BY: software. J-HX, LK, BY, and S-QL: validation. J-HX, BY, and YY: formal analysis. W-WX, JW, SG, and J-XL: investigation. S-QL and BY: resources. Z-HF and BY: data curation. J-HX and YY: writing—original draft preparation. J-HX, BY, and LK: visualization. JW and Z-HF: supervision. All authors have read and agreed to the published version of the manuscript.

## Conflict of interest

The authors declare that the research was conducted in the absence of any commercial or financial relationships that could be construed as a potential conflict of interest.

## Publisher's note

All claims expressed in this article are solely those of the authors and do not necessarily represent those of their affiliated organizations, or those of the publisher, the editors and the reviewers. Any product that may be evaluated in this article, or claim that may be made by its manufacturer, is not guaranteed or endorsed by the publisher.
